# Engineered small extracellular vesicles as a novel platform to suppress human oncovirus-associated cancers

**DOI:** 10.1186/s13027-023-00549-0

**Published:** 2023-11-01

**Authors:** Iman Owliaee, Mehran khaledian, Armin Khaghani Boroujeni, Ali Shojaeian

**Affiliations:** 1 Department of Medical Virology, Faculty of Medicine, Hamadan University of Medical Sciences, Hamadan, Iran; 2 Department of Medical Entomology, Faculty of Medicine, Hamadan University of Medical Sciences, Hamadan, Iran; 3 Skin Disease and Leishmaniasis Research Center, Isfahan University of Medical Sciences, Isfahan, Iran; 4 Research Center for Molecular Medicine, Hamadan University of Medical Sciences, Hamadan, Iran

**Keywords:** Small extracellular vesicles, Engineered Small Extracellular vesicles, Oncoviruses, Cancer

## Abstract

**Background:**

Cancer, as a complex, heterogeneous disease, is currently affecting millions of people worldwide. Even if the most common traditional treatments, namely, chemotherapy (CTx) and radiotherapy (RTx), have been so far effective in some conditions, there is still a dire need for novel, innovative approaches to treat types of cancer. In this context, oncoviruses are responsible for 12% of all malignancies, such as human papillomavirus (HPV), Merkel cell polyomavirus (MCPyV), Epstein-Barr virus (EBV), human herpesvirus 8 (HHV-8), as well as hepatitis B virus (HBV) and hepatitis C virus (HCV), and the poorest in the world also account for 80% of all human cancer cases. Against this background, nanomedicine has developed nano-based drug delivery systems (DDS) to meet the demand for drug delivery vectors, e.g., extracellular vesicles (EVs). This review article aimed to explore the potential of engineered small EVs (sEVs) in suppressing human oncovirus-associated cancers.

**Methods:**

Our search was conducted for published research between 2000 and 2022 using several international databases, including Scopus, PubMed, Web of Science, and Google Scholar. We also reviewed additional evidence from relevant published articles.

**Results:**

In this line, the findings revealed that EV engineering as a new field is witnessing the development of novel sEV-based structures, and it is expected to be advanced in the future. EVs may be further exploited in specialized applications as therapeutic or diagnostic tools. The techniques of biotechnology have been additionally utilized to create synthetic bilayers based on the physical and chemical properties of parent molecules via a top-down strategy for downsizing complicated, big particles into nano-sized sEVs.

**Conclusion:**

As the final point, EV-mediated treatments are less toxic to the body than the most conventional ones, making them a safer and even more effective option. Although many in vitro studies have so far tested the efficacy of sEVs, further research is still needed to develop their potential in animal and clinical trials to reap the therapeutic benefits of this promising platform.

## Introduction

As multicellular, heterogeneous aggregates, tumors grow uncontrollably and develop defense mechanisms against immune responses [1, 2]. In spite of significant advancements in traditional treatments for cancer, such as surgery, radiotherapy (RTx), chemotherapy (CTx), and their combination, the outcomes are still partially effective against many types of cancer, e.g., lung cancer (LC) [3, 4]. Unfortunately, CTx lacks selectivity, so healthy cells with an elevated mitotic index are also adversely impacted, resulting in undesirable side effects that can even cause or hasten death in patients living with cancer [5]. Given that types of cancer resist against traditional treatments and spread to other parts of the body, the therapies that help activate the host immune responses seem to be effective alone or in conjunction with other antineoplastic CTx drugs [1].

Genome mutations in the course of cancer typically build up over time, and then cell cycle checkpoints are improperly controlled, giving rise to this condition’s development. Such mutations mainly occur as a result of some mutagenic substances as well as the direct or indirect role of some carcinogenic viruses via their oncogenes [6]. The term *oncovirus* refers to a virus that causes cancer. Such viruses are also responsible for 12% of all malignancies. Besides, the poor world accounts for more than 80% of all human cancer cases. To date, seven types of human oncoviruses, including human papillomavirus (HPV), Merkel cell polyomavirus (MCPyV), Epstein-Barr virus (EBV), human herpesvirus 8 (HHV-8), and hepatitis B virus (HBV) and hepatitis C virus (HCV) have been recognized as the etiologic agents in this respect [7, 8]. Human oncoviruses, despite their distinct viral families, diverse tissue preferences, and various replication mechanisms, share strikingly similar pathogenic traits. These traits encompass the capacity to establish enduring persistent infections, inflict chronic damage to host cells, disrupt metabolic pathways, and elude the immune system, an ensemble of characteristics that fosters cell survival and malignant transformation [9]. In response to these challenges, the field of nanomedicine has embarked on a transformative journey. It has harnessed the potential of nano-based drug delivery systems, such as nanoparticles (NPs), liposomes (LPs), quantum dots (QDs), and dendrimers (DDs), with the explicit goal of enhancing the solubility, absorption, half-life, targeting precision, and controlled release of therapeutic agents. This branch of nanobiotechnology has also recently explored a novel avenue, using small extracellular vesicles (sEVs) as potential drug delivery vectors with both anti- and pro-inflammatory effects, depending on their specific cargo [10, 11]. These sEVs have emerged as promising candidates to overcome the limitations of existing drug delivery methods, owing to their pivotal role in intercellular communication [3]. In this field, exosomes (Exos) have been further studied as potential therapeutic platforms because of their critical involvement in disease propagation, cancer cell growth, and metastasis [12]. Indeed, the transmission and development of some diseases can be hindered by laying much focus on disease-derived Exos. As an alternative, Exos have much potential to be natural carriers for therapeutic molecules owing to their original structure and distinctive biological roles [13]. Exo-mimics, or custom-made synthetic Exos used in DDS, may correspondingly make it possible to target cancer cells by creating Exos with cell-specific targeting molecules. Exos also need to clear up many obstacles before being utilized as an efficient method of drug delivery, so it is plausible to anticipate all potential uses of such natural NPs in the future [14]. Recent research has reflected on developing engineered sEVs as a novel platform to suppress human oncovirus-associated cancers, particularly HPV, EBV, and HBV. Until now, there has been a noticeable absence of a comprehensive article addressing using exosomes to suppress human oncovirus-associated cancers. This particular aspect constitutes a novel contribution to the current paper. This review article aimed to explore the potential of engineered sEVs in suppressing human oncovirus-associated cancers, to delineate the benefits of their application as essential techniques for improving the functionality of sEVs as drug-delivery vehicles for treatment purposes, and to address the challenges to their utilization.

### EVs: biogenesis and characterization

EVs represent a diverse collection of particles, typically discharged by cells into the extracellular environment. A range of names referring to types of EVs can also be retrieved from the related literature, such as ectosomes, oncosomes, prostasomes, and microparticles (MPs) or microvesicles (MVs), originating from the plasma membrane at the range of 100–1000 nm, apoptotic bodies (ABs), as the product of dying cells once blebbing and compartmentalizing (1–5 μm), Exos, at the range of 30–100 or 150 nm, and shedding vesicles (SVs) [15–17]. In clinical settings, MVs and Exos can be discovered in various fluids, including plasma, urine, cerebrospinal fluid (CSF), amniotic fluid, bronchoalveolar lavage (BAL), synovial fluid, malignant ascites, breast milk, and saliva [18].

Over recent years, sEVs have been linked to the development some types of cancer and other diseases. Several studies have also established how sEVs contribute to cancer development [15]. Besides, EVs have been found with the potential to be utilized for drug delivery, vaccine development, and innovative diagnostic techniques. Besides, they are known as naturally-occurring cargo delivery agents that help carry viral proteins, lipids, cytokines, ribonucleic acid (RNA), and, in some circumstances, the virion itself (such as hepatitis B). This typically results in less invasiveness and mechanical stress at the site of a solid tumor, which allows learning about cellular or tissue-related states from bodily fluid samples [19, 20]. The lipid membrane of EVs can further prevent the payload from being broken down by bodily fluids, enhancing uptake via target cells [4]. To deal with cancer development, scientists have been encouraged to develop novel therapeutic delivery platforms based on EVs as nanocarriers of anti-cancer drugs with regard to their intrinsic characteristics and their role in transferring functional cargoes between cells [3]. In this respect, Exos can transport both infectious cargoes and defense-enhancing host molecules across cells, as reported, although the study of their functions in pathogen-caused infections is still in the early stages [21]. Such substances are incapable of replications, lack active nuclei, and are often enclosed by lipid bilayers. Nucleic acids, proteins, lipids, and metabolites are only a few components in EVs. Delivering EV content further urges recipient cell function reprogramming [17]. Current research also sheds light on Exos nucleic acid composition, including mitochondrial and genomic deoxyribonucleic acid (DNA) composition. Genomic areas amplified or deleted due to the presence of DNA in EVs can be thus identified, which can be single- or double-stranded (ss or ds) and ultimately reflect the cell status [16]. Besides, EVs can interact with target cells through numerous processes, viz., membrane fusion, endocytosis, phagocytosis, or cell-membrane molecules. Some molecules are also more abundant in EVs, including cytoskeletal proteins, major histocompatibility complex (MHC) class I and class II proteins, adhesion proteins (i.e., tetraspanins and integrins), heat shock proteins (HSPs), and membrane-fusion proteins (namely, Rab and annexins). These EVs are richer in tumor DNA in patients with cancer and contain high molecular weight dsDNA in terms of nucleic acids. The cytoplasm is where DNA can be transported to multivesicular bodies (MVBs) upon interacting with the tetraspanin CD63 as a protein-coding gene before being loaded into Exos, according to the proposed mechanism for DNA loading in EVs. Micronuclei, which seem unstable and often collapse to expose their content, are also considered the site of DNA loading in EVs [22]. The nucleic acids connected to EVs are highly conserved, possibly protected from oxidation by nucleases inside the lipid bilayer membranes of vesicles.

**Fig. 1:**
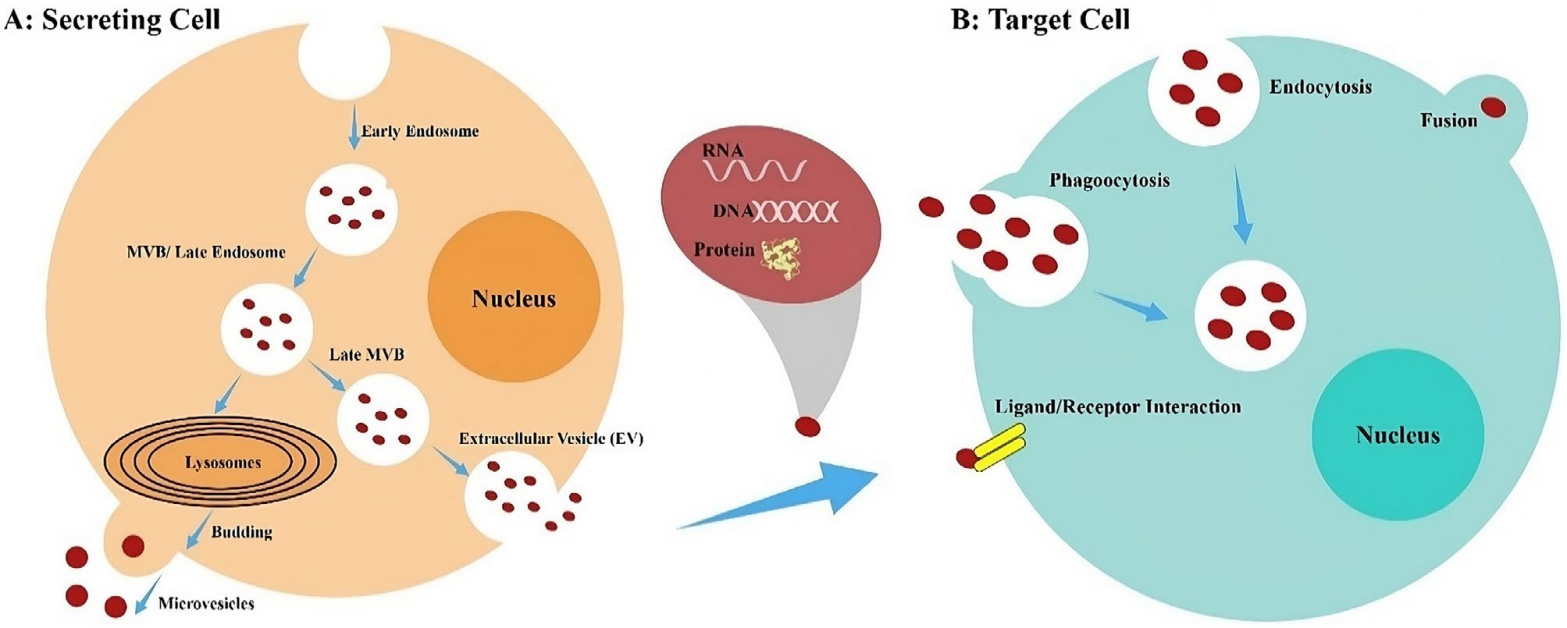
**A**: Schematic summary of EV production and genetic information transmission from one cell to another. The endosome, made up of a plasma membrane, gives rise to Exos. Inward budding also occurs as early endosomes mature into late ones, forming MVBs packed with intraluminal vesicles (ILV). Lysosomes can also break down MVBs or fuse with the membrane and release ILV, known as Exos. MVs, on the other hand, originate from the budding of the plasma membrane. **B**: Four distinct ways that Exos and MVs interact. EVs further facilitate cell-to-cell communication by transferring bioactive molecules (namely, proteins, lipids, and nucleic acids) horizontally. EVs generated by a secreting cell, such as MVs or Exos, can thus enter a target cell through fusion, endocytosis, and phagocytosis, or interact with the ligand/receptor of membrane proteins of the target cell

The blood from patients living with cancer can now be exploited for minimally invasive whole genome and whole transcriptome studies. Exosomal DNA and RNA sequencing can further identify all driver and passenger mutations, translocations, and amplifications that might serve as potential therapeutic targets [15]. Exos have also evolved into new tools for determining the host health since they mirror the content and outward appearance of the cells from which they originate. Moreover, studies using Exos as biomarkers for autoimmune diseases, types of cancer, and neurological disorders are on a rising trend [16]. Among the most intriguing applications of EVs in cancer is the potential use of such molecules as biomarkers to track the development of the disease via liquid biopsy as a non-invasive technique. Utilizing either circulating tumor cells (CTCs) or circulating tumor DNA (ctDNA), an exponential number of techniques for the minimally invasive early detection, diagnosis, and follow-up of malignancies have been accordingly developed in recent years [15]. The promise of tumor- and dendritic cell (DC)-derived Exos in cancer immunotherapy has been further highlighted in two landmark articles that established the area of Exo-based cancer therapies two decades ago. Exos are now being developed as potential novel biopharmaceuticals and vaccines for the treatment and prevention of a number of diseases. Current approaches are based on the engineering of EVs, (i) isolated from ex vivo cell cultures, (ii) purified from biological fluids (namely, plasma, serum, urine, and saliva), and (iii) produced in vitro from cultured cells [23]. Healthy, cancerous, and virus-infected cells all release Exos, which have recently been identified as a mechanism utilized by cancerous and virus-infected cells to influence their microenvironment, thereby affecting the development of surrounding cells by the intercellular transfer of various signaling molecules and viral micro (mi)RNAs [24]. Exos can further transmit information to recipient cells through three different mechanisms, i.e., (i) direct protein-protein interaction (PPI) between exosomal membrane and that of receptor cell, which initiates an intracellular signaling cascade, (ii) membrane fusion with recipient cell, releasing exosomal content into it, and (iii) direct phagocytosis of Exos by target cells, which internalizes them into their constituent parts [25] (Fig. 1). Exo production and transfer suppression by targeted therapy is thus an effective method to obstruct the spread of tumors, particularly their metastasis. Inhibiting the spread of cancer by simultaneously targeting cancer cells and Exos accordingly has a promising impact, suggesting that the given approach can one day be practiced to treat tumors [26].

### SEV engineering

As a new field, sEVs engineering has currently attracted much interest, and more novel sEV-based structures are expected to be developed in the future. The improved stability, biocompatibility, biodistribution, and pharmacokinetic properties of sEVs have been further employed to increase their effectiveness [27]. Moreover, some changes have been made to deal with uncertainties about the quick clearance of unaltered sEVs as targeted drug carriers [28]. Direct and indirect adjustments are briefly categorized into two kinds for sEVs engineering methodologies. Of note, sEVs are normally engineered to be utilized for medicinal purposes. As well, sEVs-based semisynthetic nano-vesicles have been developed by modifying sEVs cargoes to carry various endogenous or exogenous components and then change the targeting moieties [29]. Such vesicles may be utilized in specialized applications as therapeutic or diagnostic tools. The main objectives of the related studies have now been expanded to include the design and production of fully synthetic sEVs-mimic particles using biotechnology techniques, which can help overcome some production restrictions, including those associated with large-scale manufacturing, isolation, modification, and purification. The various methods applied to create synthetic sEVs are divided into two categories: (i) the creation of synthetic bilayers based on the physical and chemical properties of parent molecules, known as a bottom-up approach, and (ii) a top-down strategy for downsizing complicated, big particles into nano-sized sEVs [29]. Cancer therapy using clustered, regularly interspaced short palindromic repeats and CRISPR-associated protein 9 (CRISPR/Cas9) is also regarded as effective. Distributing CRISPR/Cas9 components in vivo in a safe and effective manner, however, is still a challenging endeavor. Thanks to their advantages in terms of biocompatibility, structural stability, restricted immunogenicity, and minimal cytotoxicity, EVs are among the excellent choices for the packaging and delivery of the CRISPR/Cas9 system for cancer treatment. As a novel method to modify gene profiles in host cells, EV-encapsulated CRISPR/Cas9 has recently come under the spotlight in new research [30].

**Fig. 2:**
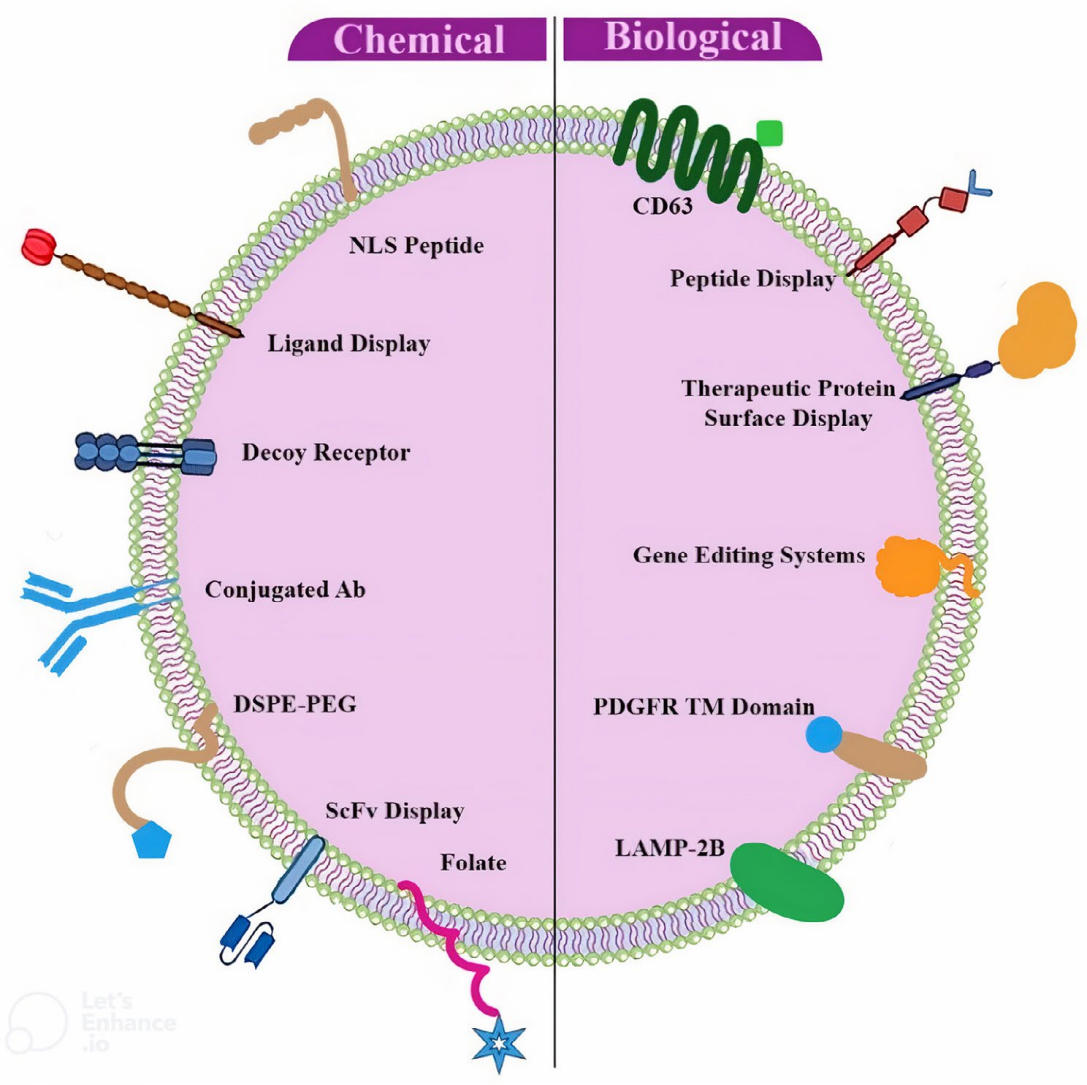
Surface engineering of Exos by genetic/biological manipulation or chemical modification. Chemical modifications install various entities, such as peptides, proteins, lipids, aptamers, small molecules, and polymers via chemical reactions or lipid-lipid interactions in lipids or membrane-bound proteins. Biological engineering accordingly introduces targeting motifs, namely, peptides and proteins, through the gene fusion of membrane-bound proteins. DSPE- PEG; 1, 2-distearoyl-sn-glycero-3-phosphoethanolamine-polyethylene glycol, scFvs; single-chain variable fragments, Ab; antibody, LAMP; lysosome-associated membrane protein, PDGFR; platelet-derived growth factor receptor

#### Direct modifications of surface proteins

Exosomal surface proteins are essential for the biodistribution and targeting of sEVs. The surface of such vesicles has been thus altered using a wide variety of techniques. The targeting capability of sEVs can be further improved by proper surface modifications. However, the reaction conditions must be closely monitored to prevent sEVs aggregation or disruption [31]. In this respect, sEVs surface engineering has been recognized to use covalent alterations as a significant strategy. Non-living vesicles are also sEVs. As a result, some substances and processes that are fatal to living cells can be utilized to create these vesicles. Moreover, covalent interactions substantially have stronger average bonds than the non-covalent ones, leading to more stable surface changes [32]. A type of surface-modified sEVs with improved targeting capability was also produced by Tian et al. (2018). On the exterior of sEVs, cyclo (Arg-Gly-Asp-DTyr-Lys) peptides were accordingly coupled with dibenzocyclooctyne (DBCO). The altered sEVs were then mixed with curcumin. The ischemic areas in the brain also received more curcumin from these modified sEVs than the non-modified ones. This engineering approach could be thus practiced to quickly produce functionalized sEVs in large quantities [33]. The active location of the proteins expressed on the surface of sEVs is still anticipated to alter as a result of covalent changes. It is thus necessary to do additional experimental evaluations of such adjustments [34]. The non-covalent methods are another technique to alter the surface of sEVs. Three main categories are also used to classify non-covalent modifications, viz., receptor-ligand binding, multivalent electrostatic interactions, and hydrophobic insertion (Fig. 2). A surface-modified sEV was further created in 2016 using the receptor-ligand approach for cancer treatment. Superparamagnetic NPs were thus attached to the surface of blood-derived sEVs in this investigation using transferrin. These NPs successfully hit the surface-mounted transferrin receptors of sEVs. The surface-modified sEVs then enhanced the targeting of cancer cells, according to in vivo studies completed under an external magnetic field [35]. Maguire et al. successfully attached biotinylated magnetic NPs to the surface of sEVs by targeting transgenic biotin-acceptor peptides on the surface of sEVs [36]. Cationic species are further being exploited in multivalent electrostatic interactions to modify sEVs. In this method, highly cationic substances cling to the negative charge of exosomal membranes. These altered sEVs have accordingly demonstrated improved cytosolic transport as well as the successful release of exosomal content into the cytosol. Using cationic lipids, it is also possible to create electrostatically engineered multivalent sEVs with improved cellular absorption [37]. Nevertheless, endocytosis, which frequently results in lysosomal breakdown, represents the way cationic lipids are typically absorbed into cells. The toxicity of these NPs to cells is another issue [38]. Hence, additional research is required to enhance multivalent electrostatic interactions. The sEV surface engineering has also looked at hydrophobic interactions. Exploiting this method, hydrophobic and lipophilic chemicals have been naturally integrated onto the surface of sEVs [39]. Due to the high cholesterol concentration in their membranes, sEVs seem stiffer than their donor cells. As a result, they typically need extreme temperatures or hostile environments for fusion. The hydrophobic interaction is also a straightforward technique that helps promote sEV fusion compared to other sEV modification practices. In this way, hydrophobic species are incubated with sEVs at a temperature between 25 and 37 °C [40]. This method has been successfully utilized to create some commercial sEVs and fill sEVs with tiny lipophilic medications such as doxorubicin, curcumin, and cucurbitacin [41–43]. Compared with other techniques, hydrophobic changes require lengthy incubation durations.

#### Indirect modification via genetic manipulation of parental cells

Genetic engineering of parental cells has been so far employed to modify exosomal membrane proteins to produce modified sEVs at cellular levels. In this method, parental cells are simply transfected using plasmids or viral vectors containing the desired genes. The transduced cells then secrete the required protein into EVs or onto their surface as a result of the presence of the coding sequences of signal peptides. Accordingly, the cell culture supernatant can be utilized to produce sEVs with the target protein [44]. This method was effectively tapped to create reporter systems by stabilizing the integration of fluorescent fusion proteins within the surface of sEVs. The parental cells, however, may impact the yield of changed sEVs and their physicochemical characteristics [45]. Moreover, the surface proteins, lipids, and indicators that constitute the surface can significantly affect how well the sEVs function. Thus, it is interesting to investigate suitable parental cell sources for sEV engineering. Likewise, small interfering (si)RNAs or micro (mi)RNAs have recently been applied to transfect parental cells [46]. For instance, myocardial ischemia injury has been avoided by sEVs made from adipose stem cells that were overexpressing miR-126. Moreover, miR-126-enriched sEVs have strongly aided in the production and migration of microvascular cells [47]. In addition, sEVs containing non-coding (nc)RNAs can control drug resistance. MiR-214 was accordingly transfected into HEK293T cells by Wang et al. The exo-anti-214 produced by HEK293T cells also has the potential to overcome cisplatin resistance in gastric cancer (GC) [48]. Just 15–20% of the sEVs edited by miRNAs accordingly had anti-epidermal growth factor receptor (EGFR) nano-bodies on their membrane, according to Kooijmans et al. (2016). This investigation demonstrated that the effectiveness of the parental cell transfection utilizing non-coding (nc)RNAs was not constant but greatly influenced by the RNA species [49]. Another difficulty is the fact that large complexes, such as protein-oligonucleotide conjugates, might encapsulate and transport ncRNAs to target cells during sEV purification [50]. A few exosomal components also promote the overexpression of ncRNAs [51]. As a result, these problems must be addressed when sEVs are genetically altered.

## Role of engineered sEVs in suppressing human oncovirus-associated cancers

### Hepatitis B virus

Hepatitis B Virus (HBV) is a member of the Hepadnaviridae family that only affects hepatocytes That is transmitted vertically and horizontally, from person to person [52]. It is currently present in almost two billion people, and is the cause of half of viral hepatitis fatalities worldwide [53–56]. With around 905,677 new cases and 830,180 cancer-associated fatalities every year, liver cancer (LC) is the sixth most common malignancy across the world. Notably, it was also the third leading cause of cancer-associated death in 2020 [57]. The most common subtype of hepatic carcinoma, hepatocellular carcinoma (HCC), also accounts for 75–85% of all primary LC cases. HCV and HBV, respectively damage the liver over time and have lately been linked to the development of HCC and carcinogenesis [58]. The three phases of chronic hepatitis B (CHB) are immune-tolerant, immune-active, and inactive. Different levels of necroinflammation, with or without fibrosis, are further seen in the liver during the immune-active phase. In this domain, antiviral therapy can help minimize hepatitis flare-ups, but liver failure significantly contributes to total morbidity, even with antiviral drugs [54].

Many drugs, including conventional and pegylated interferons (IFNs) and nucleoside analogues (NAs), have thus received approval from both the United States (US) Food and Drug Administration (FDA) and the European Medicines Agency for the treatment of chronic HBV infection in children (including lamivudine, adefovir, entecavir, and tenofovir) [59]. Even though a highly effective HBV vaccine has developed, there are still a number of problems, such as vaccination cost, non-compliance, non-responsiveness, and vaccine escape mutations [56]. Finding new drugs for targeted therapy for patients with HCC is thus crucial. As mentioned, sEVs have the unique ability to deliver functional molecules and change the biological behavior of recipient cells, which highlights their potential use as the best therapeutic vehicles for the treatment of cancer, both theoretically and practically. In order to serve as an alternative to CTx and targeted medicines, altered sEVs are currently being developed. They have many benefits over earlier DDS because of their ability to penetrate biological barriers and accomplish extremely effective drug delivery. Given that sEVs are cellular in origin, they are well tolerated and can easily evade immune clearance, which lowers drug dose and toxicity [58, 60, 61]. Studies have further shown that Exo-mediated transduction of IFN-α-induced antiviral responses from liver non-parenchymal cells (LNPCs) to HBV-infected hepatocytes might restore an antiviral state in hepatocytes. LNPCs may also use Exos containing antiviral compounds to deliver the antiviral response induced by IFN-α to hepatocytes exposed to HBV. Exos also mediate and improve IFN-anti-HBV as therapeutic actions. Comparing the Exos released by HBx-infected Huh7 cells with those released by a control one have accordingly allowed researchers to identify distinct changes in the protein contents of Exos [56]. For instance, it was discovered that Exos contributed to the antiviral responses from IFN and the transfer of HBV components. The Exos produced by Kupffer cells and other LNPCs could further facilitate the cell-to-cell transmission of HBV-specific antiviral compounds triggered by IFN [53]. Moreover, Exos and their content have been shown to aid in the human immune response induced by HBV over recent years [54]. As reported in some studies, Exo-mediated HCV and HBV infection may involve EVs and their biogenesis pathways [62]. Accordingly, an effective novel HBV prevention and treatment method may be developed since there is a better knowledge of the roles of Exos during HBV infection. Therefore, the functions of Exos in HBV include, (i) direct involvement in HBV replication, (ii) immunologic responses during HBV, (iii) potential selection of exosomal RNAs and proteins as novel biomarkers for HBV diagnosis, and (iv) development of Exos-based vaccinations [63]. To treat diseases, engineered Exos can be thus loaded with certain molecules and delivered in vivo. The elicited Exos contain significant levels of HBV core proteins following the fusion of the Exo-anchoring protein Nef mutant (Nefmut) and the HBV core proteins (Table 1). These modified Exos present a new path for the HBV vaccine candidates since the reconstitution of activated HBV-specific cytotoxic T lymphocytes (CTLs) can bring therapeutic effects. The Exos released by the HBV-infected cells also carry viral information, and may accordingly contribute to viral pathogenicity and transmission. Exos also have the ability to carry antiviral drugs and trigger antiviral responses, making them useful for the development of preventive and therapeutic vaccines. However, more studies are needed before their application for therapeutic purposes [63]. Exos have been further linked in some studies to HCV and HBV prevention [64]. In this line, Li et al. discovered that exosomal antiviral compounds from IFN-loaded LNPCs prevented HBV [65]. As well, exosomal Homo sapiens (hsa)-miR-193a-5p, hsa-miR-25-5p, and hsa-miR-574-5p have demonstrated to partially limit HBV replication and transcription, while hsa-miR-574-5p has shown to bind to the 2750–2757 location of the HBV genome sequence to lower the levels of pregenomic (pg)RNA and polymerase mRNA. In addition, Exos can transport the IFN-induced antiviral responses from LNPCs to HBV-infected hepatocytes. They can therefore enhance the therapeutic effect of IFN on HBV by regaining the hepatocyte antiviral state. For HBV diagnosis and treatment, standard criteria have already been developed, but more studies are still required to investigate novel drugs that can prevent viral multiplication and transmission, and then minimize the side effects. As a whole, Exos play a crucial role in the formation of viral particles, support virus transmission, and affect pathogenicity in HBV, as explained in this study. Another potential method for treating HBV is blocking pathways connected to Exos. Even though Exos generated from HBV are being studied, it is obvious that they can be employed in clinical practices even at the early level [59].

### Hepatitis C virus

The prevalence of chronic hepatitis C (CHC) is globally rising, with a morbidity rate of more than 3.5% in Central and East Asia and North Africa. Individuals with CHC are thus at the increased risk of cirrhosis and LC caused by the Hepatitis C Virus (HCV) [66]. Around 3% of the world’s population, or 170 million people, are now suffering from HCV. It is, thus, one of the main factors in developing chronic liver conditions, such as cirrhosis, steatosis, and hepatocellular carcinoma (HCC). Despite this, producing efficient HCV vaccines is still challenging. Among the main causes of chronic hepatitis, resulting in fibrosis, cirrhosis, and HCC, is the positive-sense single-stranded (ss)RNA virus, known as HCV [67]. In this respect, pegylated IFN-α and ribavirin are the traditional treatments for this condition. The recent advanced direct-acting antivirals, such as the protease inhibitor simeprevir and the NS sofosbuvir, have optimized therapeutic effects by increasing the sustained virological response (SVR) rate (namely, the frequency of treated patients achieving SVR) in patients with HCV. The use of sEVs generated from macrophages or umbilical cord mesenchymal stem cells (UMSCs) has also been acknowledged for HCV reduction [21]. As well, HCV Exos are a smart technique that may be employed by the virus to ensure efficient replication, and account for INF treatment resistance for this condition. These discoveries might lead to novel HCV-related Exo-targeting drugs [56]. The useful miRNAs, primarily let-7f, miR-145, miR-199a, and miR-221 released from UMSC-Exo, significantly contribute to the drop in the HCV-RNA replication (Table 1). Pegylated IFN-α and ribavirin are also two main components of the traditional treatment for HCV. Regarding their use or purpose, there are still some unresolved issues, such as virus resistance, concurrent side responses, and clearly high medical costs. Exosomal miRNAs have also had a synergistic impact once taken with commercially recognized anti-HCV medications, like IFN or telaprevir. They have mostly inhibited viral infection by targeting the viral replication stage. The normal human stem cells used to harvest and purify UMSC-Exo, which effectively prevent HCV, have had the distinct benefit of being naturally produced substances with low cytotoxicity. The addition of Exos at a late post-entry stage of viral infection, even with a lengthy incubation time, has accordingly led to the significant suppression of HCV. However, the incubation of UMSC-Exo during an early phase of the life cycle of HCV did not cause the anti-HCV effect [19]. Shrivastava et al. (2016) also showed that the Exo marker CD63 was related to an increased IFN-stimulated bone marrow stromal cell antigen 2 (BST-2) gene in autophagy knockdown cells, which might prevent HCV assembly or release. Infectious HCV particles could not be further released by Exos once the autophagy mechanism was silenced. They also noticed that the formation of HCV-infectious extracellular and intracellular particles was inhibited by Rab27a knockdown [68]. According to Bukong et al., using an Exo-targeted Ago2-miR-122-HSP90 inhibitor system could prevent the host factors from effectively modulating HCV transmission [69]. Another study published in 2017 further discovered a novel use for mast cell (MC)-derived Exos in promoting HCC cell invasion and migration. Exos produced by MC in response to HCV-E2 stimulation could yet prevent HCC cell invasion and movement. Accordingly, HCV-E2 augmented the expression of miR-490 in recipient HCC cells and MC-derived Exos, which mitigated the activity of the EGFR/AKT/ERK1/2 pathway, and prevented the migration of the HCC cells. They also established that miR-490 could be delivered by MCs to the HCC cells via Exos to block the EGFR/AKT/ERK1/2 pathway, which could in turn thwart tumor spread [70]. According to Dreux et al. Exos carrying HCV-RNA, could also cause plasmacytoid DCs (pDCs) to secrete IFN-α. In this context, IFN-α could block the viral life cycle and prevent the proliferation of viruses [71]. On the word of Giugliano et al. IFNs caused liver sinusoidal endothelial cells (LSECs) to release antiviral Exos, eventually preventing HCV replication [72]. Yet, in anti-tumor therapy, HCC cells produce Exos with increased HSPs, which promote natural killer (NK) cell anti-tumor bioactivity [56].

### Epstein-barr virus

As an oncogenic, human g-herpes virus, Epstein-Barr virus (EBV) causes human malignancies, such as B-cell lymphoma, T-cell and NK cell lymphoma, gastric cancer, nasopharyngeal carcinoma (NPC), and some autoimmune diseases [17, 73]. It has also been linked to some lymphoid and epithelial malignancies, including Burkitt lymphoma, Hodgkin’s disease, and acquired immunodeficiency syndrome (AIDS)-associated immunoblastic lymphoma. EBV is assumed responsible for about 1% of all human cancers [17, 74, 75]. A number of human cancers, namely, NPC, EBV-associated GC (EBVaGC), and specific types of lymphomas are further associated with the endemic EBV, which was first identified in 1964 [76].

Depending on the location and level of differentiation of the infected B cell, EBV-infected B cells can display four distinct gene expression patterns in vivo. The infectious viruses are thus created using one of these programs (namely, the lytic cycle). The last three are all linked to latent infection, in which no contagious virus is generated. In this vein, Epstein-Barr nuclear antigen (EBNA) and latency membrane protein (LMP) are thought to be freshly infected cells in the tonsils of healthy EBV carriers. In contrast, germinal center and memory B cells express a more constrained collection of latency proteins [74]. The expression of these genes further causes the immortalization and abnormal proliferation of B cells after infection, producing lymphoblastoid cell lines (LCLs). Clinical research has accordingly shown that the EBV1 cases in Hodgkin’s lymphoma and diffuse large B-cell lymphoma (DLBCL) in the elderly have a worse prognosis than the EBV2 ones. Therefore, developing novel therapeutic approaches that explicitly address EBV1 B-cell lymphoma seems necessary [17]. The Exos of various types, including those associated with EBV and the ones that are exclusively produced from NPC, have been similarly isolated from the serum of infected patients. Since these Exos have varied contents, they have unpredictable effects on the immune system, angiogenesis, cell proliferation, cell-to-cell communication, and tumor invasion [77]. Exos also have a role in latent viral infection, and tumor viruses like EBV and the Kaposi sarcoma-associated herpes virus (KSHV) can influence the tumor microenvironment (TME) by secreting Exos with particular viral components. The EB nuclear antigen 1 (EBNA1), latent membrane protein 1 (LMP-1), LMP-2 A, EB virus-encoded small RNAs (EBERs), and BamHI A rightward transcripts (BARTs) encoded by EBV46 are also present in the Exos produced by EBV-infected cells, also known as EBV-related Exos. Some types of cells, such as EBV-positive NPC cells or EBV-transformed LCLs, might release Exos relevant to the EBV infection [77]. In this context, Chen et al. (2022) provided new insights into EBV-associated tumors with much focus on Exos and established that EBV Exos could develop as a new biomarker or therapeutic target for EBV-associated malignancies thanks to their possible immunosuppressive effects. Exo-targeted treatment therefore can have potential applications. Exos have been used in molecularly targeted tumor therapy as new drug delivery vehicles. Moreover, Exo separation technology is gradually improving. There is currently no EBV vaccination available, and the treatments for the related malignancies have simply had sporadic success. Exo-based disease monitoring and treatment as well as viral vaccines may be thus promising in the future for the treatment and prevention of EBV malignancies, but such strategies still need more research [76]. Exos carrying a particular siRNA were functionally modified to target oncogenic KRAS9 in murine pancreatic cancer (PC) cells, according to a study by Kase et al. (2021). This suggests that the modified Exos have therapeutic potentials for malignant tumors whose molecular target has been thus far identified. In this study, they created oral squamous cell carcinoma (OSCC)-targeted Exos (octExos) that could produce a transmembrane protein (namely, EBV-induced-3 [EBI3]) on their membranes, and was abundantly expressed by the OSCC cells of the head and neck, and demonstrated that treatment with certain Exos (including octExos) could suppress the growth of oral cancer cells [78] (Table 1).

### Human papillomavirus

Smoking, drinking alcohol, and Human Papillomavirus (HPV) are among the well-known risk factors for developing oral cancer (OC). Unfortunately, there has been little progress in overall survival for patients with distant metastases, with an increase in the prevalence of oral cavity tumors over the previous few decades [79]. The papillomaviruses are a large family of non-enveloped, small DNA viruses that can cause squamous epithelial tumors (warts and papillomas) in various anatomical locations. The circular dsDNA genome of HPVs is 8 kb long. It is also organized into three main regions, viz., (i) the upstream regulatory region (URR), which is the origin of replication and contains transcription factor-binding sites to regulate gene expression, (ii) the early region (ER), which codes for six genes involved in viral replication and cell transformation (namely, E1, E2, E4, E5, E6, E7), and (iii) the late region (LR), which codes for the L1 and L2 proteins [80, 81]. Depending on their propensity to cause cervical cancer (CC), mucosal HPV types have been divided into high-risk (HR) and low-risk (LR) ones. The International Agency for Research on Cancer (IARC) also classifies twelve HR-HPV types as carcinogenic (Group 1: 16, 18, 31, 33, 35, 39, 45, 51, 52, 56, 58, and 59). In fewer patients, other genotypes, like HPV33, HPV35, and HPV58, have been further reported [80]. The oropharynx, penis, cervix, vagina, vulva, and anus are all areas where HR-HPV has been shown to cause cancer. CC also accounts for 3.4% of all cancer-related deaths worldwide. In 2020, CC led to more than 341,831 deaths in females. As well, 99.7% of cases of CC are linked to HR-HPV, primarily HPV16 and HPV18 [15, 82, 83]. The IARC has further found a significant association between HPV and cancers affecting the cervix, penis, vulva, vagina, anus, oropharynx (containing the tonsils and base of the tongue), oral cavity, larynx, and hypopharynx [81]. The oncogenic potential of HPV resides in its capacity to interfere with some key cell cycle regulators via its oncogenic proteins, E6 and E7. Moreover, HPV has been reported in various cancers, i.e., glioblastoma (GBM), colorectal, lung, and breast cancers, but its pathogenic role still remains controversial.

CC is caused by persistent infection of HR-HPV [84–86]. Due to circulating HPV-DNA in individuals with CC or precancerous lesions, research over the past several years has shown the existence of cervical HPV-DNA in EVs. The circulating HPV-DNA sequences have also been reported in patients without cervical lesions, originating from cervical cells with integrated genomes. Ambrosio et al., on the other hand, recovered plasma-derived Exos from a male patient with colon cancer and HPV type 16 integrated into the lesion site. These Exos included HPV-DNA type 16, so researchers theorized that colonic cells acquired this DNA via being exposed to Exos containing HPV-DNA. However, it was demonstrated that HVP was present in Exos generated from keratinocytes [16]. Moreover, oropharyngeal cancers (OPCs) and other HPV-associated malignancies have not yet been the subject of Exos research. Given the mounting evidence of Exo involvement in viral infection and intercellular communication, Exo content in HPV-associated malignancies warrants investigations [18]. Exos generated by regulatory T cells (Tregs) invariably contain different cargoes than those released by DCs or activated T cells. While Tregs have been mostly credited with producing immunosuppressive Exos, other cells, such as MSCs, produce Exos that assist in moderating inflammation [16]. Hence, the ability of Exos produced from HR-HPV-positive cells to promote tumors may depend on the dissemination of oncogenic miRNAs to recipient cells. The majority of the miRNAs affected by E6/E7 exhibit carcinogenic properties [15]. The chimeric Nefmut/anti-HPV16-E7 scFv product is successfully uploaded in EVs, binds HPV16-E7, and prevents the growth of cells that express HPV16-E7 [87]. MART-1, glycoprotein 100 (gp100), tyrosinase related protein-1 (TRP-1), Her2/neu, and carcinoembryonic antigen (CEA) are also examples of trans-membrane proteins as trimeric autotransporter adhesins (TAAs) that spontaneously associate with Exos, and then activate particular anti-tumor T cell responses. Exos that had been created in vitro to upload a large amount of HPV-E7 coupled with Nefmut have recently shown to trigger an anti-E7 CTL immunologic response in an effective manner once administered to mice [23] (Table 1). In addition, Wang et al. (2020) demonstrated miR-34a downregulation in malignancies with HPV positivity. Hence, substituting miR-34a may be a potential treatment for HPV-positive malignancies [10]. In 2020, Hofmann et al. explored the developing function of Exos in the head and neck cancer diagnostics, prognosis, and therapy, and found that modified Exos could be thought of as therapeutic anti-cancer vaccines for HPV-associated cancers. This strategy was suggested based on the remarkable ability of the mutant HIV-1 negative regulatory factor (viz., Nefmut) protein to integrate into Exos and function as an Exo-anchoring protein upon fusion with heterologous proteins [88]. Another study in 2022 reported that Exos from HPV-16 E7-pulsed DCs prevented the CC advancement by controlling macrophage activity, whose mechanism was pertinent to rat cationic amino acid transporter 2 (CAT2) protein [89].

The sixth most frequent cancer worldwide, head and neck SCC (HNSCC), is further distinguished by severe immune suppression. As prospective noninvasive liquid indicators, Exos have also been discovered in this domain. Exos have recently been shown to have significant potentials as liquid biomarkers in HNSCC for disease activity, tumor stage, immune suppression degree, therapeutic response, and outcomes [88]. It is common for patients with metastatic or recurrent HNSCC not to respond to standard treatments or develop drug resistance. In addition to preventing chemoresistance and cytotoxic side effects, the targeted delivery of chemotherapeutics may improve the efficacy of such treatments. Besides, siRNAs and chemotherapeutics are both being delivered through Exos, which are endogenous nanocarriers for a wide variety of compounds. In contrast to studies on HNSCC, their potential for drug administration has been investigated in a number of tumor forms, including BC and PC. Exos containing the chemotherapeutics, doxorubicin, or paclitaxel have also been demonstrated to aggregate well in target tumor tissues and reduce tumor growth in a BC-induced animal model without any adverse effects [90].

### Human T-lymphotropic virus type 1

Bloodborne human retroviruses from the genus *Deltaretrovirus* include the Human T-lymphotropic virus Type 1 (HTLV-1). Across the world, 10 million people are infected by HTLV-1, with the Caribbean, South America, Western and Southern Africa, Iran, Japan, and Australia having the highest endemicity. First discovered in 1979, HTLV-1 was initially described in 1980 [91]. As well, Orthoretrovirinae and Spumaretrovirinae are the two genera that make up the family Retroviridae. The Orthoretrovinae subfamilies also contain all known viruses that can infect humans and other animals and cause sickness. The significant human pathogens HTLV-1, HTLV-2, HTLV-3, and bovine leukemia virus (BLV) are all members of the Deltaretrovirus genus. All retroviruses generally consist of two ss-genomic (ssg)RNAs enclosed in a capsid and an envelope. Retroviruses are RNA viruses that can reverse transcribe into the host genome through an intermediary DNA molecule, which then acts as a template for viral mRNA and proteins. The majority of particles have a diameter of around 100 nm and contain viral protease, retrotranscriptase (RT), integrase (IN), and group-specific antigen (gag), including capsid, nucleocapsid, and matrix [16]. As well, 90–95% of those with HTLV-1 infection remain asymptomatic. However, adult T-cell leukemia/lymphoma (ATL), aggressive lymphoproliferative disorders, and inflammatory syndromes, such as HTLV-1-associated myelopathy or tropical spastic paraparesis (HAM/TSP) are caused by HTLV-1 [92, 93]. Although each retrovirus has a unique cellular tropism, they all infect cells by adhering to a cellular receptor to the viral envelope, fusing the membrane and viral entry. Due to the mutagenesis that results from insertions into genes or promoters, retroviruses are de facto protooncogenic since they can integrate anywhere in the human genome. Viral gene transcription can further start from the long terminal repeats (LTR) at either end of the provirus once incorporated into the host genome. Both the virus and the host exert strong control over the transcription of viral genes [16]. Tax and HTLV-1 basic leucine zipper factor (HBZ) are also possible markers for disease development among HTLV-1 regulatory and auxiliary genes due to their relationship with viral infectivity and the growth and survival of leukemic cells. Nuclear protein Tax is a highly immunogenic protein, since it alters the cell cycle and the traditional nuclear factor kappa B (NF-κB) pathway, and then increases viral transcription in the 5’LTR region. In contrast, HBZ inhibits the traditional NF-κB pathway and the transcription of viral genes having a promoter region in the 3’LTR region. The virions of the HTLV-1 are spherical and pleomorphic and range in size from 100 to 120 nm. In this line, sEVs have a similar size and biogenesis to HTLV-1 virions [92]. Researchers have also discovered that sEVs build up in the bloodstream of HTLV-1 carriers. These vesicles are particularly rich in lysosomal and mitochondrial proteins, and are most likely released from uninfected cells (or cells of hematopoietic origin, whether they are infected or not) in response to viral detection or inflammation rather than from infected cells [93].

### Kaposi sarcoma-associated herpesvirus

Many human cancers, including Kaposi’s sarcoma, primary effusion lymphoma (PEL), and multicentric Castleman’s disease (MCD), are caused by the Kaposi Sarcoma-associated Herpesvirus (KSHV) [94, 95]. The immunosuppressed, especially those with human immunodeficiency virus (HIV) infection, are thus at an increased risk of developing KSHV-associated malignancies. Patients with weak immune systems also have substantially greater morbidity due to KSHV-related illnesses than individuals with strong ones [94]. The gamma herpesvirus subfamily similarly includes KSHV. KSHV, like all herpesviruses, is also a large dsDNA virus with two distinct life cycle phases, latency and lytic (productive) replication. The viral genome is further preserved as a nuclear episome in latency, with little viral gene expression. While most viral genes are expressed, the viral genome undergoes extensive replication during lytic replication, and virions are assembled and released from cells [94, 96]. Although latently infected cells are directly linked to KSHV-associated cancers, latent and lytic infections contribute to tumorigenesis. In addition, lytic replication spreads infection and multiplies the population of latently infected cells, which later become transformed cells, creating a pro-inflammatory and proliferation environment [96]. EVs further modulate the pathogenesis, infectiousness, and immunological responses against viruses from virus-infected cells. The similar endocytic pathways between viruses and EVs may thus provide insights into new antiviral drug targets [97]. In this respect, Jeon et al. (2017) discovered that complement activation could encourage KSHV chronic latent infection by activating the NF-κB pathway, which could improve the survival of KSHV-infected cells and prevent viral lytic replication. They further identified the underlying mechanism of complement activation by EVs and a unique function of EVs generated by KSHV during de novo infection [98]. Based on the proteins reported by Meckes et al. (2013), it was predicted that KSHV and EBV Exos shared a number of characteristics. Thus, cancer is a predicted disease, cell death, and survival are predicted to be the commonly affected cellular function, and eukaryotic initiation factor-2 (eif2) signaling is to be the commonly affected canonical pathway. Recent research has further demonstrated that KSHV can change the metabolism of the host B-cells to favor glycolysis, and the Exos produced by KSHV-infected cells are substantially enriched in proteins that are often involved in glycolysis [99].

### Merkel cell polyomavirus

A rare, aggressive neuroendocrine form of skin cancer (SC) with a high mortality rate is known as Merkel cell carcinoma (MCC). Many immunosuppressed patients thus have a higher risk of getting MCC. The viral DNA of Merkel cell polyomavirus (MCPyV) is present in about 80% of all MCCs [100]. As there is currently no effective systemic therapy for this particular type of cancer, an Exo-based therapy is typically suggested [101]. Small T-antigen (ST) and large T-antigen (LT) are further encoded by the polyomavirus early region (ER), while late structural proteins, VP1 and VP2, are present in the LR [102]. There is currently no effective systemic therapy for this particular type of cancer, but surgical excision and postoperative RTx [101]. Each polyomavirus, including MCV, is a non-enveloped, dsDNA virus with a circular genome that contains ER and LR on opposing strands and a non-coding control region (ncCR) in the middle that houses the viral origin of replication [103]. Moreover, antigen-presenting cells (APCs) can recognize Exos, and cell-to-cell-mediated immune activation results in anti-tumor responses. Exo-based vaccinations have been further produced to treat cancer and examine the potential contribution of MCC-produced Exos to carcinogenesis. An Exo-based therapy suggested for this type of cancer currently lacks efficient systemic treatment [101]. The involvement of miR-375 in intercellular communication between MCC tumor cells and stromal fibroblasts was established by Fan et al. (2021). They showed that endogenously produced miR-375 from MCC cells could cause fibroblast polarization in vitro, and this process could also come about in vivo in MCC patients. These findings imply that miR-375 is a desirable target for therapeutic interventions; in fact, the therapeutic viability of targeting miR-155 in cutaneous T-cell lymphoma is now being explored [104] (Table 1).

**Table 1:** Summarizes the engineered sEVs and their clinical applications

Virus	Engineered sEVs	Clinical Application	References
**HBV**	sEVs delivering IFN-α from LNPCs	HBV-infected hepatocytes might restore an antiviral state in hepatocytes.	(56)
	Exo-anchoring protein Nef mutant (Nefmut)	Activated HBV-specific cytotoxic T lymphocytes (CTLs) can bring therapeutic effects.	(63)
	Exosomal Homo sapiens (hsa)-miR-193a-5p, hsa-miR-25-5p, and hsa-miR-574-5p	Limit HBV replication and transcription	(59)
**HCV**	sEVs loaded with anti-HCV miRNAs (let-7f, miR-145, miR-199a, and miR-221)	Suppression of HCV replication	(19)
	Exo marker CD63 was related to increased IFN-stimulated bone marrow stromal cell antigen 2 (BST-2) gene in autophagy knockdown cells	which might prevent HCV assembly or release.	(68)
	Exo-targeted Ago2-miR-122-HSP90 inhibitor system	could prevent the host factors from effectively modulating HCV transmission	(69)
**EBV**	sEVs delivering specific siRNA	functionally modified to target oncogenic KRAS9 in murine pancreatic cancer (PC) cells	(78)
**HPV**	chimeric Nefmut/anti-HPV16-E7	Binds to HPV16-E7 and prevents the growth of cells that express HPV16-E7	(87)
	MART-1, gp100, TRP-1, Her2/neu, and CEA spontaneously associate with Exos.	Activate particular anti-tumor T cell responses.	(23)
	miR-34a	Downregulation in malignancies with HPV	(10)
	Exos from HPV-16 E7-pulsed DCs	prevented cervical cancer advancement by controlling macrophage activity in rat CAT2 protein	(89)
**HTLV-1**	No clinically relevant applications have been reported yet	Potential biomarkers and therapies still under investigation	(91)
**KSHV**	sEVs carrying viral miRNAs (miRK12-3-5p, miR-K12-2-5p, mir-10b-5p, mir-143-3p)	Can change the metabolism of the host B-cells to favor glycolysis	(99)
**MCPyV**	sEVs carrying the miR-375 and viability of targeting miR-155	A desirable target for therapeutic interventions in cutaneous T-cell lymphoma is now being explored	(104)

## Preclinical evidence supporting sEVs for drug delivery

A BC-induced mouse model was subcutaneously injected with sEVs harboring miR-let-7a, and this treatment demonstrated an anti-cancer effect via targeting EGFR [105]. Some studies have further confirmed the value of sEVs in delivering drugs encapsulated in EVs to treat HCC [106]. An innovative method for producing the inducible caspase 9 (iCasp9) suicide gene in engineered sEVs-associated adeno-associated viruses has also been described. In an in vivo xenograft model, the modified sEVs increased HCC regression because they were low in immunogenicity and toxicity, and were easily absorbed by the HCC cells [107]. A study also encapsulated erastin and rose bengal into sEVs and engineered CD47 on the surface to protect them from phagocytosis by macrophages. The sEVs induced obvious ferroptosis in HCC, with minimized toxicity in the liver and kidneys. In view of this, previous studies have developed NPs targeting specific adhesion or receptor proteins on the surface of sEVs membranes for targeted drug delivery. In this respect, Tian et al. (2018) designed a nano-drug based on the PDCM (a type of NP that consists of four components) system by targeting sEVs-shuttling miR-21 and miR-10b, which markedly diminished HCC growth and the number of metastatic lung nodules [58]. High purity and sufficient output are thus required for sEVs technology to function as a drug delivery vector. Nevertheless, the effectiveness of the existing approaches seems to be limited by a number of issues, including the length of time, high cost, and production of toxic byproducts. The generation of intracellular calcium, external stress, cytoskeletal blockage, pharmacological stimulation, along the induction of gene expression factors are all methods used by oncologists to boost the overall yield of sEVs [58].

## Challenges and opportunities for small extracellular vesicles

While sEVs show promising potential as drug delivery vectors, several challenges must still be addressed for them to come to market realistically. Perhaps the greatest hurdle is the lack of standardized, scalable production methods required for clinical and commercial applications [108]. Current isolation techniques are slow, inefficient, and can produce impure sEVs preparations of inconsistent quality [51]. This hinders characterization, dose optimization, and quality control efforts needed for regulatory approval. Genetic engineering of parental cells is another barrier, as it remains difficult to reliably produce large batches of sEVs with homogeneous, targeted modifications [44]. Additional challenges include developing robust stability studies, understanding sEVs clearance kinetics, and ensuring minimal toxicity and immunogenicity, which require substantial fundamental and preclinical research [27]. However, the opportunities are also vast if these obstacles can be overcome. Improved production and consistency could facilitate the clinical translation of sEVs therapeutics while surmounting engineering hurdles may unlock sEVs’ full potential as customizable drug delivery systems. With further progress in fundamental sEVs sciences and enabling technologies, the future of this cutting-edge platform in disease treatment looks bright indeed [109].

## Conclusion and discussion

Cancer is known as a complex and heterogeneous disease that affects millions of people worldwide. Although the most common traditional treatments, such as CTx and RTx, have been thus far effective in some cases, there is still a need for innovative approaches to treat cancer. One such approach drawing much attention in recent years is using sEVs as a novel platform for delivering therapeutics [109]. More recently, engineered sEVs have become an increasingly attractive platform for suppressing human oncovirus-associated cancers. According to a recent study by Mahmoudvand et al. (2022) [110], EVs can be exploited to efficiently transfer genetic materials into cancer cells, potentially suppressing oncoviruses. Furthermore, EVs are capable of delivering DNA, siRNAs, short hairpin RNA or small hairpin RNA (shRNA), and miRNAs to cancer cells in a safe and effective manner, making them highly appealing for the development of gene therapy. These siRNAs bind to and degrade the viral RNA, thus preventing the virus from replicating and reducing the likelihood of tumor formation. As such, EVs are able to modulate disease pathogenesis in types of infections, such as HIV-1, HTLV-1, Ebola virus (EBOV), and Rift Valley fever (RVF) by promoting cell-to-cell spread, immune evasion, persistent inflammation, and cell-cycle regulation in recipient cells [19]. Oxytetracycline, approved by the US FDA, could further minimize the generation of EVs that contain the Ebola VP40 in order to limit EBOV infection and safeguard the adaptive immune system [111].

Previous studies have also demonstrated that EVs produced from symbiotic vaginal lactobacilli contain antiviral components, including host restriction factor (HRF) mRNAs, and have specifically improved resistance to HIV reproduction and transmission [21]. Low immunogenicity, good biosecurity, and a potent ability to penetrate solid tissues are among the advantages of EVs. As a result, it is a type of nano-engineering system frequently utilized in drug delivery to prevent viral infection and the onset of disease. The developed miRNA-401 targeting HSV-1 ICP4 mRNA is also packaged into engineered sEVs and given to virally sensitive cells, which can provide an antiviral environment for at least 72 h to more effectively inhibit the replication of HSV-1. To target these infected cells exclusively, an antibody (Ab) specific for particular viruses, such as HIV and the HPV, can be packed inside the manufactured EVs [111]. Furthermore, many configurations of the complex Ab structure can be decreased without compromising the binding effectiveness. One of the smallest molecules with antigen-binding capability is the single-chain variable fragment (scFv). Targeting intracellular pathogenic factors by engineered sEVs with scFvs accordingly has the potential to be practiced as a revolutionary treatment strategy [87]. Exo-based drug delivery has also been researched for many years. In this line, Kase et al. (2021) showed that fibroblast cell Exos could be used to accomplish precise targeting with anti-tumor effects [78]. Cells that express ligands with high binding affinities for the target cells may also produce Exos. Nevertheless, coating liposomes with a functionalized polymer increases targeted drug delivery by creating nano-bins. In addition to being essential for intracellular communication, EVs might be a brand-new, intrinsic antiviral defense mechanism. Viral infection, replication, dissemination, and pathogenesis are all promoted by viruses by using the EVs biogenesis route. Over and above enhancing their pathogenesis, viruses control antiviral immune responses [112]. Furthermore, EVs can be engineered to be more biocompatible and have a longer life span in the body, implying that they are able to remain in the body for a longer period of time and provide sustained protection against cancer. Finally, EV-mediated treatments are less toxic to the body than the traditional ones, making them a safer and more effective option. While many studies have to date tested sEVs efficacy in vitro, further research is still needed to develop the potential of sEVs in animal and clinical trials to reap the therapeutic benefits of this promising platform [82]. Another approach to utilizing engineered sEVs for cancer therapy is to load them with CRISPR-Cas9 gene-editing tools, which can be programmed to target and disrupt specific viral genes, thus preventing the virus from replicating and reducing the likelihood of tumor formation [30]. The lack of optimal purification methods for the isolation of Exos with high purity, dosage optimization, measurement standards, and administration routes, adverse effects when used in combination with therapeutic cargoes, and immunogenicity of heterogeneous components due to the nature of the donor cells are among the limitations of using Exos. Given such concerns, the quality control (QC) of Exos should be carefully performed. Moreover, the biological fate of Exos and their effects on target organs should be fully understood. Despite these obstacles, using Exos in various diseases, including cancers, seems attractive. In particular, oncovirus-associated cancers inspire the future clinical development of these biomarkers.

## Future perspective

This review highlights the nascent but rapidly advancing field of engineered sEVs to suppress human oncovirus-associated cancers. While still early in development, proactive foresight into the potential future applications of this technology can guide research and accelerate clinical translation. In the near-term, priority should be given to optimizing sEVs production methods, cargo loading, and tumor-targeting moieties. Standardized protocols and good manufacturing practices will need to be established to enable generation of clinical-grade sEVs. Combinations with standard chemotherapy, radiation, and immunotherapies should be explored. Advanced modification techniques may allow precision engineering of designer sEVs with multiple functionalities. These include environment-responsive triggers for controlled drug release, co-delivery of synergistic drug combinations, and built-in imaging reporters to track sEVs fate in real-time. Synthetic biology approaches could also recreate sEVs properties. The future looks bright for this emerging biotechnology toward improving patient outcomes. While the applications of engineered sEVs for suppressing human oncovirus-associated cancers are still in the early stages of development. Further research is thus needed to optimize sEVs as a drug delivery platform and evaluate their safety and efficacy in preclinical and clinical studies.

## Data Availability

Data sharing is not applicable to this article as no datasets were generated or analysed during the current study.

## Abbreviations

HPV: Human Papillomavirus
MCPyV: Merkel cell polyomavirus
EBV: Epstein-Barr virus
HHV-8: Human Herpesvirus 8
HBV: Hepatitis B virus
HCV: Hepatitis C virus
DDS: Drug delivery systems
sEVs: Small extracellular vesicles
MVs: Microvesicles
ABs: Apoptotic bodies
Exos: Exosomes
CTCs: Circulating tumor cells
ctDNA: Circulating tumor DNA
ENVs: sEV-based semisynthetic nanovesicles
DBCO: Dibenzocyclooctyne
EGFR: Epidermal growth factor receptor
HCC: Hepatocellular carcinoma
CHB: Chronic hepatitis B
LNPCs: Liver nonparenchymal cells
Nefmut: Nef mutant
CHC: Chronic hepatitis C
MC: Mast cell
SVR: Sustained virological response
UMSC: Umbilical cord mesenchymal stromal cells
pDCs: Plasmacytoid dendritic cells
NPC: Nasopharyngeal carcinoma
EBVaGC: EBV-associated gastric cancer
EBNA: Epstein-Barr nuclear antigen
LMP: Latency membrane protein
LCLs: Lymphoblastoid cell lines
DLBCL: Diffuse large B-cell lymphoma
KSHV: Kaposi sarcoma-associated herpesvirus
octExos: OSCC-targeted exosomes
SCC: Squamous cell carcinoma
URR: Upstream regulatory region
HR: High-risk
LR: Low-risk
NSCC: Head and neck squamous cell carcinoma
siRNAs: Small interfering RNAs
HTLV-1: Human T-cell lymphotropic virus type 1
BLV: Bovine leukemia virus
RT: Retrotranscriptase
IN: Integrase
ATL: Adult T-cell leukemia/lymphoma
HAM/TSP: HTLV-1-associated myelopathy or tropical spastic paraparesis
LTR: Long terminal repeat
HBZ: HTLV-1 basic leucine zipper factor
NF-κB: Nuclear factor kappa B
PEL: Primary effusion lymphoma
MCD: Multicentric Castleman’s disease
HIV: Human immunodeficiency virus
MCC: Merkel cell carcinoma
sT: Small T-antigen
LT: Large T-antigen
NCCR: Non-coding control region
APCs: Antigen-presenting cells
shRNA: Short hairpin RNA
FDA: Food and Drug Administration
HRFs: Host restriction factors
scFv: Single-chain variable fragment
QC: Quality control
iCasp9: Inducible caspase 9
PDCM: 1-palmitoyl-2-oleoyl-sn-glycero-3-phosphocholine,1,2-dioleoyl-sn-glycero-3-phosphoethanolamine, cholesteryl hemisuccinate, and 2-[4-(morpholinyl)ethyl]-4-oxobutanoate

## References

[1] Garofalo M, Villa A, Rizzi N, Kuryk L, Rinner B, Cerullo V et al. Extracellular vesicles enhance the targeted delivery of immunogenic oncolytic adenovirus and paclitaxel in immunocompetent mice. J Control Release [Internet]. 2019;294:165–75. 10.1016/j.jconrel.2018.12.022. 10.1016/j.jconrel.2018.12.022 30557650

[2] Mogheri F, Jokar E, Afshin R, Akbari AA, Dadashpour M, Firouzi-amandi A et al. Co-delivery of metformin and silibinin in dual-drug loaded nanoparticles synergistically improves chemotherapy in human non-small cell lung cancer A549 cells. J Drug Deliv Sci Technol [Internet]. 2021;66(January):102752. 10.1016/j.jddst.2021.102752.

[3] Jayasinghe MK, Tan M, Peng B, Yang Y, Sethi G, Pirisinu M et al. New approaches in extracellular vesicle engineering for improving the efficacy of anti-cancer therapies. Semin Cancer Biol [Internet]. 2021;74(November 2020):62–78. 10.1016/j.semcancer.2021.02.010. 10.1016/j.semcancer.2021.02.010 33609665

[4] Garofalo M, Saari H, Somersalo P, Crescenti D, Kuryk L, Aksela L et al. Antitumor effect of oncolytic virus and paclitaxel encapsulated in extracellular vesicles for lung cancer treatment. J Control Release [Internet]. 2018;283:223–34. 10.1016/j.jconrel.2018.05.015. 10.1016/j.jconrel.2018.05.015 29864473

[5] O’Brien MER, Borthwick A, Rigg A, Leary A, Assersohn L, Last K (2006). Mortality within 30 days of chemotherapy: a clinical governance benchmarking issue for oncology patients. Br J Cancer.

[6] Seyed-Khorrami S-M, Soleimanjahi H, Łos MJ, Zandi K, Emameh RZ (2023). Oncolytic viruses as emerging therapy against cancers including Oncovirus-induced cancers. Eur J Pharmacol.

[7] Saravanan C, Baskar M, Ahmed SSSJ, Veerabathiran R. Role of viral human oncogenesis: recent developments in molecular approaches. Oncog Viruses. 2023;147:–72.

[8] Aga M, Bentz GL, Raffa S, Torrisi MR, Kondo S, Wakisaka N (2014). Exosomal HIF1α supports invasive potential of nasopharyngeal carcinoma-associated LMP1-positive exosomes. Oncogene.

[9] Tornesello ML, Cerasuolo A, Starita N, Tornesello AL, Bonelli P, Tuccillo FM (2022). The Molecular Interplay between Human oncoviruses and Telomerase in Cancer Development. Cancers (Basel).

[10] Sadri Nahand J, Moghoofei M, Salmaninejad A, Bahmanpour Z, Karimzadeh M, Nasiri M (2020). Pathogenic role of exosomes and microRNAs in HPV-mediated inflammation and Cervical cancer: a review. Int J Cancer.

[11] Javan N, Khadem Ansari MH, Dadashpour M, Khojastehfard M, Bastami M, Rahmati-Yamchi M (2019). Synergistic Antiproliferative effects of Co-nanoencapsulated Curcumin and Chrysin on MDA-MB-231 Breast Cancer cells through upregulating miR-132 and miR-502c. Nutr Cancer.

[12] Soltani S, Mansouri K, Emami Aleagha MS, Moasefi N, Yavari N, Shakouri SK (2022). Extracellular vesicle therapy for type 1 Diabetes. Front Immunol.

[13] Li X, Corbett AL, Taatizadeh E, Tasnim N, Little JP, Garnis C (2019). Challenges and opportunities in exosome research-perspectives from biology, engineering, and cancer therapy. APL Bioeng.

[14] Bandopadhyay M, Bharadwaj M. Exosomal miRNAs in hepatitis B virus related liver disease: a new hope for biomarker. Gut Pathog [Internet]. 2020;12(1):23. 10.1186/s13099-020-00353-w. 10.1186/s13099-020-00353-w PMC7183117 32346400

[15] Guenat D, Hermetet F, Prétet JL, Mougin C (2017). Exosomes and other extracellular vesicles in HPV transmission and carcinogenesis. Viruses.

[16] Anderson M, Kashanchi F, Jacobson S (2018). Role of exosomes in human retroviral mediated disorders. J Neuroimmune Pharmacol.

[17] Higuchi H, Yamakawa N, Imadome KI, Yahata T, Kotaki R, Ogata J (2018). Role of exosomes as a proinflammatory mediator in the development of EBV-associated Lymphoma. Blood.

[18] Principe S, Hui ABY, Bruce J, Sinha A, Liu FF, Kislinger T (2013). Tumor-derived exosomes and microvesicles in Head and Neck cancer: implications for Tumor biology and biomarker discovery. Proteomics.

[19] Kim Y, Mensah GA, Sharif S, Al, Pinto DO, Branscome H, Yelamanchili SV (2021). Extracellular vesicles from infected cells are released prior to virion release. Cells.

[20] Metzner C, Zaruba M. On the relationship of viral particles and extracellular vesicles: implications for viral vector technology. Viruses. 2021;13(7). 10.3390/v13071238 PMC8310354 34206771

[21] Qian X, Xu C, Fang S, Zhao P, Wang Y, Liu H (2016). Exosomal MicroRNAs derived from umbilical mesenchymal stem cells inhibit Hepatitis C virus Infection. Stem Cells Transl Med.

[22] Mulcahy LA, Pink RC, Carter DRF. Routes and mechanisms of extracellular vesicle uptake. J Extracell Vesicles. 2014;3. 10.3402/jev.v3.24641 PMC4122821 25143819

[23] Di Bonito P, Accardi L, Galati L, Ferrantelli F, Federico M. Anti-cancer vaccine for HPV-associated Neoplasms: focus on a therapeutic HPV vaccine based on a novel Tumor antigen delivery method using endogenously engineered exosomes. Cancers (Basel). 2019;11(2). 10.3390/cancers11020138 PMC6406600 30682811

[24] Polakovicova I, Jerez S, Wichmann IA, Sandoval-Bórquez A, Carrasco-Véliz N, Corvalán AH (2018). Role of microRNAs and exosomes in Helicobacter pylori and Epstein-Barr virus associated gastric cancers. Front Microbiol.

[25] Hanjani NA, Esmaelizad N, Zanganeh S, Gharavi AT, Heidarizadeh P, Radfar M et al. Emerging role of exosomes as biomarkers in cancer treatment and diagnosis. Crit Rev Oncol Hematol [Internet]. 2022;169:103565. Available from: https://www.sciencedirect.com/science/article/pii/S1040842821003528. 10.1016/j.critrevonc.2021.103565 34871719

[26] Yang E, Wang X, Gong Z, Yu M, Wu H, Zhang D. Exosome-mediated metabolic reprogramming: the emerging role in tumor microenvironment remodeling and its influence on cancer progression. Signal Transduct Target Ther [Internet]. 2020;5(1):1–13. 10.1038/s41392-020-00359-5. 10.1038/s41392-020-00359-5 PMC7572387 33077737

[27] Luan X, Sansanaphongpricha K, Myers I, Chen H, Yuan H, Sun D. Engineering exosomes as refined biological nanoplatforms for drug delivery. Acta Pharmacol Sin [Internet]. 2017;38(6):754–63. 10.1038/aps.2017.12. 10.1038/aps.2017.12 PMC5520184 28392567

[28] Bunggulawa EJ, Wang W, Yin T, Wang N, Durkan C, Wang Y et al. Recent advancements in the use of exosomes as drug delivery systems. J Nanobiotechnology [Internet]. 2018;16(1):81. 10.1186/s12951-018-0403-9. 10.1186/s12951-018-0403-9 PMC6190562 30326899

[29] García-Manrique P, Gutiérrez G, Blanco-López MC. Fully Artificial Exosomes: Towards New Theranostic Biomaterials. Trends Biotechnol [Internet]. 2018;36(1):10–4. Available from: https://www.sciencedirect.com/science/article/pii/S0167779917302676. 10.1016/j.tibtech.2017.10.005 29074309

[30] Yan B, Liang Y. New therapeutics for Extracellular vesicles: delivering CRISPR for Cancer Treatment. Vol. 23, Int J Mol Sci. 2022. 10.3390/ijms232415758 PMC9779094 36555398

[31] Antimisiaris SG, Mourtas S, Marazioti A. Exosomes and Exosome-Inspired Vesicles for Targeted Drug Delivery. Vol. 10, Pharmaceutics. 2018. 10.3390/pharmaceutics10040218 PMC6321407 30404188

[32] Oude Blenke E, Klaasse G, Merten H, Plückthun A, Mastrobattista E, Martin NI. Liposome functionalization with copper-free “click chemistry.” J Control Release [Internet]. 2015;202:14–20. Available from: https://www.sciencedirect.com/science/article/pii/S0168365915000772. 10.1016/j.jconrel.2015.01.027 25626085

[33] Tian T, Zhang H-X, He C-P, Fan S, Zhu Y-L, Qi C et al. Surface functionalized exosomes as targeted drug delivery vehicles for cerebral ischemia therapy. Biomaterials [Internet]. 2018;150:137–49. Available from: https://www.sciencedirect.com/science/article/pii/S0142961217306403. 10.1016/j.biomaterials.2017.10.012 29040874

[34] Takahashi Y, Nishikawa M, Shinotsuka H, Matsui Y, Ohara S, Imai T et al. Visualization and in vivo tracking of the exosomes of murine melanoma B16-BL6 cells in mice after intravenous injection. J Biotechnol [Internet]. 2013;165(2):77–84. Available from: https://www.sciencedirect.com/science/article/pii/S0168165613001648. 10.1016/j.jbiotec.2013.03.013 23562828

[35] Qi H, Liu C, Long L, Ren Y, Zhang S, Chang X et al. Blood Exosomes Endowed with Magnetic and Targeting Properties for Cancer Therapy. ACS Nano [Internet]. 2016;10(3):3323–33. 10.1021/acsnano.5b06939. 10.1021/acsnano.5b06939 26938862

[36] Maguire CA, Balaj L, Sivaraman S, Crommentuijn MHW, Ericsson M, Mincheva-Nilsson L et al. Microvesicle-associated AAV Vector as a Novel Gene Delivery System. Mol Ther [Internet]. 2012;20(5):960–71. Available from: https://www.sciencedirect.com/science/article/pii/S1525001616319360. 10.1038/mt.2011.303 PMC3345986 22314290

[37] Nakase I, Futaki S (2015). Combined treatment with a pH-sensitive fusogenic peptide and cationic lipids achieves enhanced cytosolic delivery of exosomes. Sci Rep.

[38] Sahay G, Alakhova DY, Kabanov AV. Endocytosis of nanomedicines. J Control Release [Internet]. 2010;145(3):182–95. Available from: https://www.sciencedirect.com/science/article/pii/S0168365910002075. 10.1016/j.jconrel.2010.01.036 PMC2902597 20226220

[39] Batrakova EV, Kim MS. Using exosomes, naturally-equipped nanocarriers, for drug delivery. J Control Release [Internet]. 2015;219:396–405. Available from: https://www.sciencedirect.com/science/article/pii/S0168365915300420. 10.1016/j.jconrel.2015.07.030 PMC4656109 26241750

[40] Vázquez-Ríos AJ, Molina-Crespo Á, Bouzo BL, López-López R, Moreno-Bueno G, de la Fuente M. Exosome-mimetic nanoplatforms for targeted cancer drug delivery. J Nanobiotechnology [Internet]. 2019;17(1):85. 10.1186/s12951-019-0517-8. 10.1186/s12951-019-0517-8 PMC6637649 31319859

[41] Smyth T, Kullberg M, Malik N, Smith-Jones P, Graner MW, Anchordoquy TJ. Biodistribution and delivery efficiency of unmodified tumor-derived exosomes. J Control Release [Internet]. 2015;199:145–55. Available from: https://www.sciencedirect.com/science/article/pii/S0168365914008104. 10.1016/j.jconrel.2014.12.013 PMC4441346 25523519

[42] Harp D, Driss A, Mehrabi S, Chowdhury I, Xu W, Liu D et al. Exosomes derived from endometriotic stromal cells have enhanced angiogenic effects in vitro. Cell Tissue Res [Internet]. 2016;365(1):187–96. 10.1007/s00441-016-2358-1. 10.1007/s00441-016-2358-1 PMC4917586 26841879

[43] Zhuang X, Xiang X, Grizzle W, Sun D, Zhang S, Axtell RC et al. Treatment of Brain Inflammatory Diseases by Delivering Exosome Encapsulated Anti-inflammatory Drugs From the Nasal Region to the Brain. Mol Ther [Internet]. 2011;19(10):1769–79. Available from: https://www.sciencedirect.com/science/article/pii/S152500161632768X. 10.1038/mt.2011.164 PMC3188748 21915101

[44] Hall J, Prabhakar S, Balaj L, Lai CP, Cerione RA, Breakefield XO. Delivery of Therapeutic Proteins via Extracellular Vesicles: Review and Potential Treatments for Parkinson’s Disease, Glioma, and Schwannoma. Cell Mol Neurobiol [Internet]. 2016;36(3):417–27. 10.1007/s10571-015-0309-0. 10.1007/s10571-015-0309-0 PMC4860146 27017608

[45] Whiteside TL. Chapter Four - Tumor-Derived Exosomes and Their Role in Cancer Progression. In: Makowski GSBT-A in CC, editor. Elsevier; 2016. p. 103–41. Available from: https://www.sciencedirect.com/science/article/pii/S0065242315300056. 10.1016/bs.acc.2015.12.005 PMC5382933 27117662

[46] Cooper JM, Wiklander PBO, Nordin JZ, Al-Shawi R, Wood MJ, Vithlani M et al. Systemic exosomal siRNA delivery reduced alpha-synuclein aggregates in brains of transgenic mice. Mov Disord [Internet]. 2014;29(12):1476–85. 10.1002/mds.25978. 10.1002/mds.25978 PMC4204174 25112864

[47] Luo Q, Guo D, Liu G, Chen G, Hang M, Jin M. Exosomes from MiR-126-Overexpressing Adscs Are Therapeutic in Relieving Acute Myocardial Ischaemic Injury. Cell Physiol Biochem [Internet]. 2017;44(6):2105–16. Available from: https://www.karger.com/DOI/10.1159/000485949. 10.1159/000485949 29241208

[48] Wang X, Zhang H, Bai M, Ning T, Ge S, Deng T et al. Exosomes Serve as Nanoparticles to Deliver Anti-miR-214 to Reverse Chemoresistance to Cisplatin in Gastric Cancer. Mol Ther [Internet]. 2018;26(3):774–83. Available from: https://www.sciencedirect.com/science/article/pii/S152500161830008X. 10.1016/j.ymthe.2018.01.001 PMC5910674 29456019

[49] Kooijmans SAA, Aleza CG, Roffler SR, van Solinge WW, Vader P, Schiffelers RM. Display of GPI-anchored anti-EGFR nanobodies on extracellular vesicles promotes tumour cell targeting. J Extracell Vesicles [Internet]. 2016;5(1):31053. 10.3402/jev.v5.31053. 10.3402/jev.v5.31053 PMC4793259 26979463

[50] Lötvall J, Hill AF, Hochberg F, Buzás EI, Di Vizio D, Gardiner C et al. Minimal experimental requirements for definition of extracellular vesicles and their functions: a position statement from the International Society for Extracellular Vesicles. J Extracell Vesicles [Internet]. 2014;3(1):26913. 10.3402/jev.v3.26913. 10.3402/jev.v3.26913 PMC4275645 25536934

[51] Tkach M, Théry C. Communication by Extracellular Vesicles: Where We Are and Where We Need to Go. Cell [Internet]. 2016;164(6):1226–32. Available from: https://www.sciencedirect.com/science/article/pii/S0092867416300575. 10.1016/j.cell.2016.01.043 26967288

[52] Gholizadeh O, Akbarzadeh S, Moein M, Yasamineh S, Hosseini P, Afkhami H et al. The role of non-coding RNAs in the diagnosis of different stages (HCC, CHB, OBI) of hepatitis B infection. Microb Pathog [Internet]. 2023;176:105995. Available from: https://www.sciencedirect.com/science/article/pii/S0882401023000281. 10.1016/j.micpath.2023.105995 36681203

[53] Wu W, Wu D, Yan W, Wang Y, You J, Wan X (2021). Interferon-Induced macrophage-derived exosomes mediate antiviral activity against Hepatitis B Virus through miR-574-5p. J Infect Dis.

[54] Chen J, Xu Q, Zhang Y, Zhang H (2020). RNA profiling analysis of the serum exosomes derived from patients with chronic hepatitis and acute-on-chronic Liver Failure caused by HBV. Sci Rep.

[55] Jung S, Jacobs KFK, Shein M, Schütz AK, Mohr F, Stadler H et al. Efficient and reproducible depletion of Hepatitis B virus from plasma derived extracellular vesicles. J Extracell Vesicles. 2020;10(2). 10.1002/jev2.12040 PMC7754750 33363711

[56] Wang J, Cao D, Yang J (2020). Exosomes in Hepatitis B virus transmission and related immune response. Tohoku J Exp Med.

[57] Sung H, Ferlay J, Siegel RL, Laversanne M, Soerjomataram I, Jemal A (2021). Global Cancer statistics 2020: GLOBOCAN estimates of incidence and Mortality Worldwide for 36 cancers in 185 countries. CA Cancer J Clin.

[58] Yang S, Wang J, Wang S, Zhou A, Zhao G, Li P. Roles of small extracellular vesicles in the development, diagnosis and possible treatment strategies for hepatocellular carcinoma (review). Int J Oncol. 2022;61(2). 10.3892/ijo.2022.5381 PMC9262158 35674180

[59] Liu Z, Li Y, Wang Y, Bai X, Zhang Y (2023). Exosomes in HBV Infection. Clin Chim Acta.

[60] Ha D, Yang N, Nadithe V. Exosomes as therapeutic drug carriers and delivery vehicles across biological membranes: current perspectives and future challenges. Acta Pharm Sin B [Internet]. 2016;6(4):287–96. Available from: https://www.sciencedirect.com/science/article/pii/S2211383515301003. 10.1016/j.apsb.2016.02.001 PMC4951582 27471669

[61] Yang Y, Hong Y, Cho E, Kim GB, Kim I-S (2018). Extracellular vesicles as a platform for membrane-associated therapeutic protein delivery. J Extracell Vesicles.

[62] Sukriti S, Choudhary MC, Maras JS, Sharma S, Thangariyal S, Singh A (2019). Extracellular vesicles from Hepatitis B patients serve as reservoir of Hepatitis B virus DNA. J Viral Hepat.

[63] Li S, Li S, Wu S, Chen L. Exosomes Modulate the Viral Replication and Host Immune Responses in HBV Infection. 2019;2019. 10.1155/2019/2103943 PMC6558633 31275965

[64] Cai S, Cheng X, Pan X, Li J (2017). Emerging role of exosomes in liver physiology and pathology. Hepatol Res.

[65] Li J, Liu K, Liu Y, Xu Y, Zhang F, Yang H (2013). Exosomes mediate the cell-to-cell transmission of IFN-α-induced antiviral activity. Nat Immunol.

[66] Zhang C, Yang X, Qi Q, Gao Y, Wei Q, Han S (2018). LncRNA-HEIH in serum and exosomes as a potential biomarker in the HCV-related hepatocellular carcinoma. Cancer Biomarkers.

[67] Kim OK, Nam Deun, Hahn YS (2021). The pannexin 1/Purinergic receptor P2 × 4 pathway controls the secretion of MicroRNA-Containing exosomes by HCV-Infected hepatocytes. Hepatology.

[68] Shrivastava S, Devhare P, Sujijantarat N, Steele R, Kwon Y-C, Ray R (2016). Knockdown of Autophagy inhibits infectious Hepatitis C Virus Release by the Exosomal Pathway. J Virol.

[69] Bukong TN, Momen-Heravi F, Kodys K, Bala S, Szabo G. Exosomes from Hepatitis C infected patients transmit HCV Infection and contain replication competent viral RNA in Complex with Ago2-miR122-HSP90. PLoS Pathog. 2014;10(10). 10.1371/journal.ppat.1004424 PMC4183590 25275643

[70] Xiong L, Zhen S, Yu Q, Gong Z (2017). HCV-E2 inhibits hepatocellular carcinoma Metastasis by stimulating mast cells to secrete exosomal shuttle microRNAs. Oncol Lett.

[71] Dreux M, Garaigorta U, Boyd B, Décembre E, Chung J, Whitten-Bauer C (2012). Short-range exosomal transfer of viral RNA from infected cells to plasmacytoid dendritic cells triggers innate immunity. Cell Host Microbe.

[72] Giugliano S, Kriss M, Golden-Mason L, Dobrinskikh E, Stone AEL, Soto-Gutierrez A et al. Hepatitis C virus infection induces autocrine interferon signaling by human liver endothelial cells and release of exosomes, which inhibits viral replication. Gastroenterology [Internet]. 2015;148(2):392–402.e13. 10.1053/j.gastro.2014.10.040. 10.1053/j.gastro.2014.10.040 PMC4765499 25447848

[73] Ahmed W, Philip PS, Tariq S, Khan G. Epstein-Barr virus-encoded small RNAs (EBERs) are present in fractions related to exosomes released by EBV-transformed cells. PLoS ONE. 2014;9(6). 10.1371/journal.pone.0099163 PMC4045842 24896633

[74] Canitano A, Venturi G, Borghi M, Ammendolia MG, Fais S (2013). Exosomes released in vitro from Epstein-Barr virus (EBV)-infected cells contain EBV-encoded latent phase mRNAs. Cancer Lett.

[75] Teow S-YY, Liew K, Khoo AS-BB, Peh S-CC (2017). Pathogenic role of exosomes in epstein-barr virus (EBV)-associated cancers. Int J Biol Sci.

[76] Chen W, Xie Y, Wang T, Wang L. New insights into Epstein–Barr virus–associated tumors: Exosomes (review). Oncol Rep. 2022;47(1). 10.3892/or.2021.8224 PMC8600424 34779497

[77] Zhou Y, Xia L, Lin J, Wang H, Oyang L, Tan S (2018). Exosomes in nasopharyngeal carcinoma. J Cancer.

[78] Kase Y, Uzawa K, Wagai S, Yoshimura S, Yamamoto JI, Toeda Y et al. Engineered exosomes delivering specific tumor-suppressive RNAi attenuate oral cancer progression. Sci Rep [Internet]. 2021;11(1):1–12. 10.1038/s41598-021-85242-1. 10.1038/s41598-021-85242-1 PMC7960743 33723306

[79] Leung LL, Riaz MK, Qu X, Chan J, Meehan K (2021). Profiling of extracellular vesicles in Oral cancer, from transcriptomics to proteomics. Semin Cancer Biol.

[80] Taberna M, Mena M, Pavón MA, Alemany L, Gillison ML, Mesía R (2017). Human papillomavirus-related oropharyngeal cancer. Ann Oncol.

[81] Morshed K, Polz-Gruszka D, Szymañski M, Polz-Dacewicz M (2014). Human papillomavirus (HPV) - structure, epidemiology and pathogenesis. Otolaryngol Pol.

[82] Alkhilaiwi F, Yuan H (2022). Detection of HPV RNA in Extracellular vesicles from neuroendocrine Cervical Cancer cells. Viruses.

[83] Qiu JJ, Sun SG, Tang XY, Lin YY, Hua KQ (2020). Extracellular vesicular Wnt7b mediates HPV E6-induced Cervical cancer angiogenesis by activating the β-catenin signaling pathway. J Exp Clin Cancer Res.

[84] De Carolis S, Storci G, Ceccarelli C, Savini C, Gallucci L, Sansone P (2019). HPV DNA associates with Breast Cancer malignancy and it is transferred to Breast Cancer stromal cells by Extracellular vesicles. Front Oncol.

[85] Bhat A, Yadav J, Thakur K, Aggarwal N, Chhokar A, Tripathi T et al. Transcriptome analysis of cervical cancer exosomes and detection of HPVE6*I transcripts in exosomal RNA. BMC Cancer [Internet]. 2022;22(1):1–16. 10.1186/s12885-022-09262-4. 10.1186/s12885-022-09262-4 PMC8840784 35148692

[86] Kaczmarek M, Baj-Krzyworzeka M, Bogucki Ł, Dutsch-Wicherek M (2022). HPV-Related Cervical Cancer and Extracellular vesicles. Diagnostics.

[87] Ferrantelli F, Arenaccio C, Manfredi F, Olivetta E, Chiozzini C, Leone P (2019). The intracellular delivery of anti-HPV16 E7 scFvs through engineered extracellular vesicles inhibits the proliferation of HPV-infected cells. Int J Nanomedicine.

[88] Hofmann L, Ludwig S, Vahl JM, Brunner C, Hoffmann TK, Theodoraki MN (2020). The emerging role of exosomes in diagnosis, prognosis, and therapy in Head and Neck cancer. Int J Mol Sci.

[89] Zhang G, Liao Y, Pan X, Zhang X (2022). Exosomes from HPV-16 E7-pulsed dendritic cells prevent the migration, M1 polarization, and inflammation of macrophages in Cervical cancer by regulating catalase 2 (CAT2). Ann Transl Med.

[90] Liu C, Gao H, Lv P, Liu J, Liu G (2017). Extracellular vesicles as an efficient nanoplatform for the delivery of therapeutics. Hum Vaccin Immunother.

[91] Pinto DO, Al Sharif S, Mensah G, Cowen M, Khatkar P, Erickson J et al. Extracellular vesicles from HTLV-1 infected cells modulate target cells and viral spread. Retrovirology [Internet]. 2021;18(1):1–27. 10.1186/s12977-021-00550-8. 10.1186/s12977-021-00550-8 PMC7901226 33622348

[92] de La-Roque DGL, Santos EV, Rodrigues ES, da Costa PNM, Brauer VS, Almeida F et al. The expression of tax and HBZ genes in serum-derived extracellular vesicles from HTLV-1 carriers correlates to Proviral load and inflammatory markers. Front Microbiol. 2022;13(May). 10.3389/fmicb.2022.881634 PMC9108699 35586867

[93] Jeannin P, Chaze T, Giai Gianetto Q, Matondo M, Gout O, Gessain A (2018). Proteomic analysis of plasma extracellular vesicles reveals mitochondrial stress upon HTLV-1 Infection. Sci Rep.

[94] Barrett L, Dai L, Wang S, Qin Z (2021). Kaposi’s sarcoma-associated herpesvirus and extracellular vesicles. J Med Virol.

[95] McNamara RP, Chugh PE, Bailey A, Costantini LM, Ma Z, Bigi R et al. Extracellular vesicles from Kaposi Sarcoma-associated herpesvirus lymphoma induce long-term endothelial cell reprogramming [Internet]. Vol. 15, PLoS Pathogens. 2019. 10.1371/journal.ppat.1007536. 10.1371/journal.ppat.1007536 PMC6361468 30716130

[96] Gong D, Dai X, Xiao Y, Du Y, Chapa TJ, Johnson JR et al. Virus-like vesicles of Kaposi’s Sarcoma-Associated Herpesvirus Activate Lytic replication by triggering differentiation signaling. J Virol. 2017;91(15). 10.1128/JVI.00362-17 PMC5651724 28515293

[97] Jeon H, Kang SK, Lee MJ, Park C, Yoo SM, Kang YH (2021). Rab27b regulates extracellular vesicle production in cells infected with Kaposi’s sarcoma-associated herpesvirus to promote cell survival and persistent Infection. J Microbiol.

[98] Jeon H, Yoo SM, Choi HS, Mun JY, Kang HG, Lee J (2017). Extracellular vesicles from KSHV-infected endothelial cells activate the complement system. Oncotarget.

[99] Meckes DG, Gunawardena HP, Dekroon RM, Heaton PR, Edwards RH, Ozgur S (2013). Modulation of B-cell exosome proteins by gamma herpesvirus Infection. Proc Natl Acad Sci U S A.

[100] Konstantinell AGV. Biomarkers Discovery: the benefit of the study exosomes originated from Merkel Cell Carcinoma Cell Lines. 2019.

[101] Konstantinell A, Bruun J, Olsen R, Aspar A, Škalko-Basnet N, Sveinbjørnsson B (2016). Secretomic analysis of extracellular vesicles originating from polyomavirus‐negative and polyomavirus‐positive Merkel cell carcinoma cell lines. Proteomics.

[102] Yang R, Lee EE, Kim J, Choi JH, Chen Y, Crewe C et al. Human Polyomavirus-Encoded Circular RNAs. bioRxiv. 2020;2012–20.

[103] Ahmed MM, Cushman CH, DeCaprio JA (2022). Merkel cell polyomavirus: oncogenesis in a stable genome. Viruses.

[104] Fan K, Spassova I, Gravemeyer J, Ritter C, Horny K, Lange A (2021). Merkel cell carcinoma-derived exosome-shuttle miR-375 induces fibroblast polarization by inhibition of RBPJ and p53. Oncogene.

[105] Marleau AM, Chen CS, Joyce JA, Tullis RH (2012). Exosome removal as a therapeutic adjuvant in cancer. J Transl Med.

[106] Xue D, Han J, Liu Y, Tuo H, Peng Y (2021). Current perspectives on exosomes in the diagnosis and treatment of hepatocellular carcinoma. Cancer Biol Ther.

[107] Khan N, Maurya S, Bammidi S, Jayandharan GR (2020). AAV6 vexosomes mediate robust Suicide gene delivery in a murine model of hepatocellular carcinoma. Mol Ther Clin Dev.

[108] Lener T, Gimona M, Aigner L, Börger V, Buzas E, Camussi G (2015). Applying extracellular vesicles based therapeutics in clinical trials - an ISEV position paper. J Extracell Vesicles.

[109] Pucci C, Martinelli C, Ciofani G (2019). Innovative approaches for cancer treatment: current perspectives and new challenges. Ecancermedicalscience.

[110] Mahmoudvand S, Shokri S, Nakhaie M, Jalilian FA, Mehri-Ghahfarrokhi A, Yarani R et al. Small extracellular vesicles as key players in cancer development caused by human oncogenic viruses. Infect Agent Cancer [Internet]. 2022;17(1):1–16. 10.1186/s13027-022-00471-x. 10.1186/s13027-022-00471-x PMC9703759 36437456

[111] Yang L, Li J, Li S, Dang W, Xin S, Long S et al. Extracellular vesicles regulated by viruses and antiviral strategies. Front Cell Dev Biol. 2021;9(October). 10.3389/fcell.2021.722020 PMC8566986 34746122

[112] Caobi A, Nair M, Raymond AD. Extracellular Vesicles in the Pathogenesis of Viral Infections in Humans. Vol. 12, Viruses. 2020. 10.3390/v12101200 PMC7589806 33096825

